# Endoscopic Combined Snare-Forceps Technique for Removing Flat Sessile Polyps

**DOI:** 10.51894/001c.6349

**Published:** 2017-12-19

**Authors:** Mark W. Jones, Werner Henning

**Affiliations:** 1 McLaren Greater Lansing, Department of General Surgery, Program Director, Lansing, MI; 2 McLaren Greater Lansing, Department of General Surgery, PGY3 Resident, Lansing, MI

**Keywords:** forceps, snare, polyps, endoscopy

## Abstract

**CONTEXT:**

Current endoscopes have limitations during use in polypectomies. Specifically, polyps that are flat, broad-based and sessile are more difficult to resect. Routine polypectomy procedures allow one endoscopic device to be used at a time limiting the endoscopist. More advanced scopes are not readily available at smaller community hospitals, limiting the endoscopist to using the resources available to them.

**METHODS:**

The modification of the standard polypectomy method described here employs both an endoscopic forceps and an endoscopic snare to be used simultaneously during colonoscopy with a single lumen colonoscope. The forceps is introduced into the endoscope so the head is just projecting from the distal end of the scope. The snare is then placed just proximal to the head of the forceps outside of the endoscope. The endoscope is reinserted into the colon until the polyp is reached. Using the snare the polyp is elevated and then the snare secured around the base.

**RESULTS:**

This resulted in easier, faster, and more complete removal of flat sessile and poorly located pedunculated polyps on the first try. This technique has been employed successfully in over 20 patients at our institution.

**CONCLUSIONS:**

This new method adds another technique for endoscopists when presented with difficult polypectomies.

## INTRODUCTION

Colorectal polyps are abnormal growths found as a protuberance in the colonic lumen resulting from overgrowth of the epithelial lining of the mucosa.^1^ These can arise anywhere within the gastrointestinal tract, and have the potential of malignant transformation resulting in colorectal cancer.

Polyps are classically categorized into non-neoplastic (i.e., hyperplastic, inflammatory, mucosal, and hamartomatous (normal tissue growing in a disorganized mass) polyps and neoplastic (i.e., adenomas).^1^ Adenomas are considered precursors to colorectal cancer. It is believed that more than 80% of colon cancers arise from adenomas.^1^ The prevalence of adenomas is 25% at age 50, nearly 50% at age 70, and continues to increase with age.^1^

Common risk factors for developing adenomas include increasing age, increased body mass index, African American ethnicity, male sex, lack of physical activity, and a history of smoking.^1^ Seldom, these can develop secondary to hereditary syndromes like familial adenomatous polyposis syndrome and hereditary non-polyposis colorectal cancer.^1^ Polyps can be further classified on endoscopy based on their appearance. They can be pedunculated, meaning they arise from a stalk. A sessile polyp is attached by a broad base, typically without a stalk.^2^

The presentation of polyps also varies. Some polyps can be found incidentally during a screening colonoscopy, while other polyps present with clinical signs and symptoms such as bleeding, obstruction, intussusception, or tenesmus (i.e., the sense of having to have a bowel movement).^1^ Currently, the most effective screening procedure is the colonoscopy.^2^

A colonoscope is an instrument that providers use to directly visualize the entire colon starting at the anus and ending at the cecum. This procedure can be performed both by General Surgeon and Gastroenterologist depending on the availability of the specialist in that region. At the time of colonoscopy, one has the ability to resect and biopsy any lesions identified during the examination. There are two types of biopsy instruments commonly used. A forceps-grasping device is an instrument used to either biopsy or completely remove small polyps.^1^ A snare device is an instrument with a wire loop, which can be tightened around the base of a polyp.^1^ Once it is closed around the polyp, one can transect the polyp with this device.^1^ The majority of polyps can be removed using these two devices; however, some polyps can pose a significant challenge.

Certain types of colonic polyps can present a challenge during endoscopy. These include polyps with broad bases, flat sessile polyps, and polypoid lesions located within colonic folds. In the case of large sessile polyps, it usually is not feasible to remove the entire polyp with endoscopic forceps, and it is often challenging to encircle the base with an endoscopic snare. This results in removal of a polyp in fragments, which does not ensure complete excision and is relatively time-consuming.

Incomplete resection of the polyp is undesirable, unless surgical resection is planned. If an incomplete polypectomy is performed, the residual tissue may undergo malignant transformation.^1^ It may also lead to incorrect diagnosis as many early colon cancers are located deep within the polyp that was not removed.^3,4^ Polyps that cannot undergo complete resection will be referred for a surgical consultation. Most of these patients ultimately will undergo a colon resection.^5^

Numerous innovative techniques have been devised to facilitate easier polypectomy in these difficult scenarios.^6^ Submucosal injection with saline has proven effective in raising flat lesions to allow better access to the base of the polyp for snare removal.^7^ A suction maneuver combined with saline injection has also been described. After submucosal saline is instilled, the endoscope is positioned over the polyp and suction is applied. The scope is then slightly withdrawn temporarily tenting the polyp, which affords better access to its base. Other techniques employing double-channel scopes, endo-loops with snares, and hot avulsion procedures have also been described.^7–10^

The authors have found that the introduction of endoscopic forceps through a single-lumen colonoscope along with an endoscopic snare secured to the distal portion of the forceps externally led to easier and more complete removal of flat, broad-based, sessile polyps. The forceps is used to tent the polyp away from the colon wall. While the snare is opened, the secured forceps grasping the polyp “directs” the snare down to the targeted area. The open snare is then placed around the base of the lesion for polypectomy.

## METHODS

**Figure 1:**
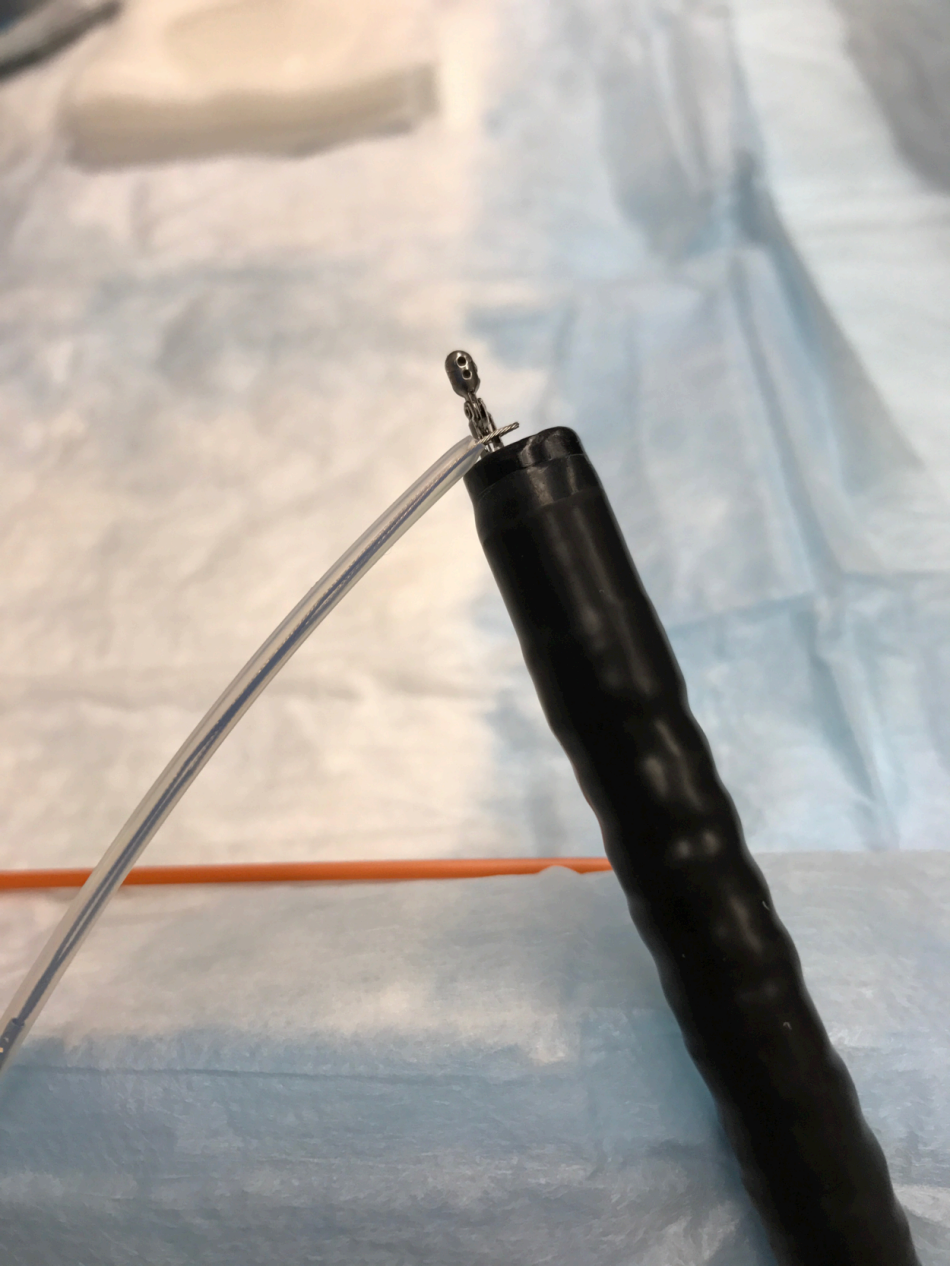
Demonstration of the Endoscopic Snare Secured to the End of the Endoscopic Forceps.

During this procedure, a standard single-lumen colonoscope is used. Once the lesion is located during colonoscopy, the scope is then removed. The forceps is introduced into the endoscope so the head is just projecting from the distal end of the scope. The snare is then placed just proximal to the head of the forceps outside of the endoscope. (Figure 1) The jaws of the forceps must be allowed to open and close freely. The endoscope is then reinserted and carefully advanced through the colon until the desired polyp is reached.

Care must be taken to ensure the tip of the endoscope with the slightly protruding forceps is kept centered in the lumen of the colon to prevent mucosal injury or perforation. This maneuver will pull the endoscopic snare along with the scope to the target. The process of performing the colonoscopy with withdrawal and reinsertion adds familiarity of the anatomy of that particular patient’s colon. This in turn makes it easier to keep the scope centered in the lumen.

**Figure 2:**
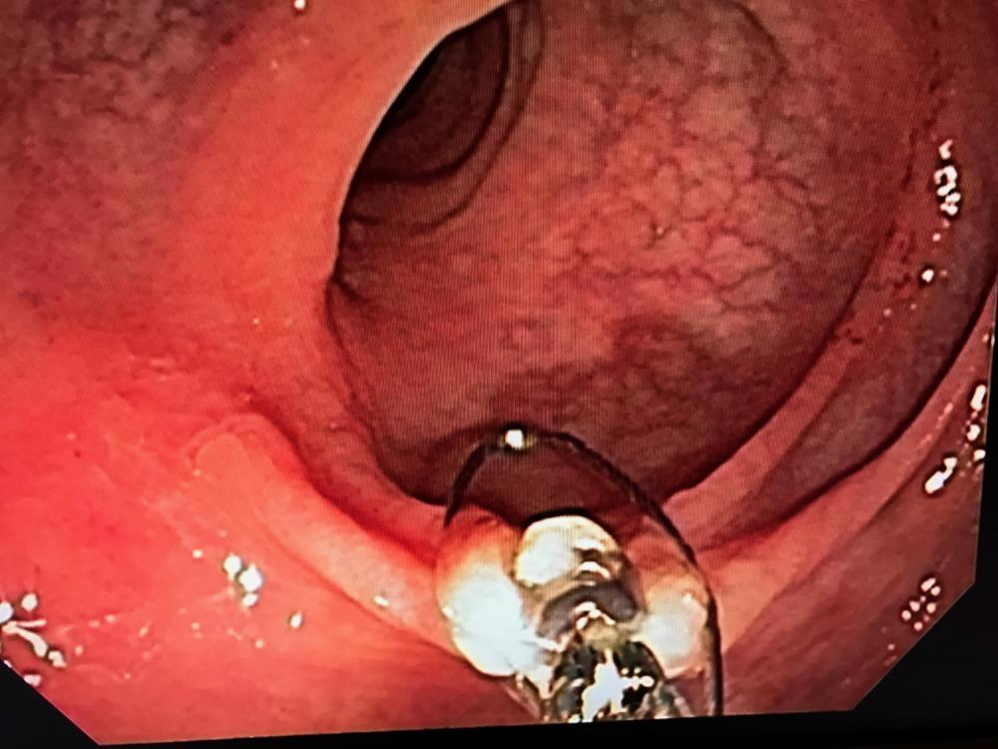
The Snare Being Placed at the Base of the Polyp as the Lesion is Tented Using the Forceps.

Once the target is reached, the forceps is advanced and used to grasp the polyp. The lesion is then tented away from the bowel wall. The endo-snare is then opened, slid down the forceps and placed around the base of the polyp. (Figure 2) Care must be taken to avoid including too much depth of the colon wall with the specimen to avoid perforation.

The authors have found this technique to be useful in removing large pedunculated polyps located behind folds or in areas difficult to access. The insertion technique is the same. The polyps are grasped with the forceps and pulled into the endoscopist’s field. This provides better visualization of the lesion’s base to ensure complete removal. The author just recently had a patient with a one-centimeter polyp hidden behind a colonic fold in the sigmoid colon. Using this technique, the author was successful in elevating the polyp out from behind the fold and securing the snare around the base. This resulted in complete resection of the polyp without great difficulty.

## RESULTS

The described technique has proven to be very effective in our practice for difficult polypectomies. We have been able to implement this technique successfully in over 20 polypectomies at our institution. Not only have the authors found it to allow for more complete resection of polyps, it has led to an easier and faster resection as well. To date, we have not experienced any complications with this technique; however, this method does have some limitations.

This method is less effective when attempted for more proximally located polyps or polyps in tortuous colons. A tortuous colon can be defined as a colon with many sharp turns and bends making it difficult to navigate through. Advancement to the targeted area may be more difficult and good judgment must be used when performing this maneuver. Care must also be taken to ensure that the externally located snare wire advances easily with the scope and does not form a bow string configuration at bends as this could injure the colon or prevent progression of the endoscope. If this does occur, the snare can be secured externally farther up the scope with a silk suture, which may lessen the bowstring effect.

It was also suggested to secure the snare directly to the tip of the endoscope, therefore eliminating the risks associated with advancing the scope with the slightly exposed forceps. This proved unsuccessful, as the snare could not be advanced over the forceps.

## CONCLUSIONS

While colonoscopies are valuable for colorectal screening, current colonoscopes have disadvantages. Most standard scopes are equipped with a single working lumen. There are more advanced scopes with two working lumens but these aren’t usually readily available in a community hospital setting. A major disadvantage is the inability to use more than one endoscopic instrument at a time.

This simple technique requires no additional training or special modifications to a single lumen endoscope. It has shown improved efficacy in complete removal of both flat, broad based sessile polyps and poorly accessible pedunculated polyps. Use of this technique could potentially prevent colectomy for incomplete polypectomy, and avoid the unnecessary morbidity and mortality of undergoing a formal colonic resection.

### Conflict of Interest

The authors declare no conflict of interest.
